# Young people's priorities for the self‐management of distress after stoma surgery due to inflammatory bowel disease: A consensus study using online nominal group technique

**DOI:** 10.1111/hex.14009

**Published:** 2024-03-10

**Authors:** Benjamin Saunders, Kay Polidano, Lucy Bray, Tamsin Fisher, Nadia Corp, Megan McDermott‐Hughes, Adam D. Farmer, Beth Morris, Sahara Fleetwood‐Beresford, Carolyn A. Chew‐Graham

**Affiliations:** ^1^ Keele School of Medicine Keele University Staffordshire UK; ^2^ Department of Sociology University of Malta Msida Malta; ^3^ Faculty of Health, Social Care and Medicine Edge Hill University Ormskirk UK; ^4^ University Hospitals of North Midlands NHS Trust Stoke‐on Trent UK; ^5^ Department of Public Health, Policy and Systems University of Liverpool Merseyside UK

**Keywords:** distress, inflammatory bowel disease, nominal group technique, self‐management, stoma surgery, young people

## Abstract

**Introduction:**

The aim of this study was to gain consensus among young people with a stoma due to inflammatory bowel disease (IBD) on the priorities for the content of an intervention for the self‐management of stoma‐related distress. The current identification and management of distress in young people with a stoma is often suboptimal in clinical settings and there is a need for improved support resources.

**Methods:**

Two consensus group meetings were carried out via online video conferencing, using nominal group technique. Participants generated, rated on a Likert scale and discussed, topics for inclusion in a future self‐management intervention.

**Results:**

Nineteen young people, aged 19–33, with a stoma due to IBD took part in one of two group meetings. Participants were located across England, Scotland, and Northern Ireland. Twenty‐nine topics were generated by participants, seven of which reached consensus of ≥80%, that is, a mean of ≥5.6 on a 7‐point Likert scale. These were: receiving advice from young people with lived experience of stoma surgery; advice on/addressing concerns about romantic relationships, sex and intimacy; information about fertility and pregnancy related to stoma surgery; stoma ‘hacks’, for example, useful everyday tips regarding clothing, making bag changes easier and so forth; reflecting on and recognising own emotional response to surgery; tips on managing the stoma during the night; and processing trauma related to the illness and surgery journey.

**Conclusions:**

Findings extend previous research on young people's experiences of stoma surgery, by generating consensus on young people's priorities for managing distress related to surgery and living with a stoma. These priorities include topics not previously reported in the literature, including the need for information about fertility and pregnancy. Findings will inform the development of a self‐management resource for young people with an IBD stoma and have relevance for the clinical management of stoma‐related distress in this population.

**Patient or Public Contribution:**

Three patient contributors are co‐authors on this paper, having contributed to the study design, interpretation of results and writing of the manuscript. The study's Patient and Public Involvement and Engagement advisory group also had an integral role in the study. They met with the research team for four 2‐h virtual meetings, giving input on the aims and purpose of the study, recruitment methods, and interpretation of findings. The group also advised on the age range for participants. The views of young people with a stoma are the central component of the study reported in this paper, which aims to gain consensus among young people with an IBD stoma on their priorities for the content of a resource to self‐manage distress related to stoma surgery.

## 1 INTRODUCTION

Approximately 21,000 people a year in the United Kingdom undergo surgery to remove all, or part of, their large bowel,^1^ resulting in them having a stoma; an opening in the abdomen through which faeces are collected in a bag attached to the skin (for individuals with Crohn's disease, small bowel resection can also result in stoma formation). While some individuals have a temporary stoma, which can later be reversed through further surgery, for others their stoma is permanent, and therefore lifelong. Stoma surgery is often a result of inflammatory bowel disease (IBD), a group of inflammatory conditions affecting the gastrointestinal tract, the most common of which are ulcerative colitis (UC) and Crohn's disease. Both conditions give rise to similar symptoms, including abdominal pain, vomiting, bloody diarrhoea, and weight loss.

While stoma surgery can result in positive outcomes for individuals with IBD through relieving symptoms,^2^, ^3^ impact on quality of life and experiencing distress is common.^4^, ^5^ In this context, we consider distress as a broad spectrum of emotional or psychological responses that includes worries and concerns that may be transitory but which can also persist over time.^6^ Stoma‐related distress can result from body image concerns,^7^ sexual difficulties,^8^ reduced social functioning^9^ and lowered self‐esteem.^10^ Individuals who undergo stoma surgery are at increased risk of developing depression or anxiety.^11^

Stoma surgery is a possibility faced by many young adults, aged 16–35 (from here on referred to collectively as ‘young people’), as stoma surgery is highest in the first 10 years after IBD diagnosis^12^ and IBD is commonly diagnosed from late adolescence to mid‐20s.^13^ Stoma‐related distress may also be particularly hard to manage for people in this age group, who are also undergoing major life transitions, such as moving out of the family home, going to college/university or beginning full‐time employment.^14^ Early identification and effective management of distress is crucial for improving young people's quality of life, and can reduce the possibility that distress will persist or worsen, and potentially lead to a diagnosis of depression.^15^ However, our previous qualitative research with young people with an IBD stoma and healthcare professionals (HCPs) who treat these patients (general practitioners, IBD nurses, stoma nurses, gastroenterologists, colorectal surgeons) showed that distress is often not discussed and/or is suboptimally managed in clinical settings.^3^, ^16^

It is therefore important that there are evidence‐based interventions and resources to improve the management of, and reduce, distress in this population. While this can include interventions to improve the management of stoma‐related distress within healthcare settings, interventions focused on supporting young people with an IBD stoma to self‐manage distress may be particularly beneficial,^17^ given the increased pressure on healthcare provision across primary and secondary care services. Additionally, the young people in our previous study reported the desire for age‐appropriate online resources to support self‐management of stoma‐related distress; however, such resources do not currently exist.^3^, ^16^ Encouraging individuals with long‐term conditions to self‐manage their health conditions is part of the UK National Health Service Long Term Plan, with the aim to ‘support and empower people to manage their ongoing physical and mental health conditions themselves’.^18^

To address this need, in the current National Institute for Health Research (NIHR)‐funded Stoma Support Study, we are co‐designing with young people and HCPs an intervention to support the identification and management of stoma‐related distress in young people, aged 16–35. The proposed intervention will be in two parts: (1) an online resource to help young people to self‐manage distress; and (2) a training package for HCPs involved in the care of young people with a stoma. This paper focuses on part 1 of the planned intervention, the self‐management resource. Part 2 is the focus of a separate, linked paper.^19^

The first step in developing the self‐management resource was to establish the topic areas that are most important to young people. This study aimed to gain consensus among young people with an IBD stoma on their priorities for the content of a resource to self‐manage distress related to stoma surgery.

## 2 METHODS

### 2.1 Study design

Nominal group technique (NGT) was used, which is a consensus group method that follows a systematic approach to building consensus through structured meetings.^20^ NGT follows a set of stages that involves participants initially generating topics on a particular issue within the meeting and sharing these topics with the wider group, following which each participant individually rates each topic. The results of the ratings are discussed among the group, and topics that are not agreed upon can then be rerated individually, with participants given the opportunity to change their scores based on the group discussions.^20^ We aimed to include a geographically diverse group of participants, and it was therefore decided to adapt the NGT method for online data collection, with meetings hosted on Microsoft Teams® video‐conferencing platform. Consensus group meetings were video‐recorded using the Microsoft Teams recording function. The use of online meetings to develop consensus has been successful in another recent study developing a health intervention.^21^ The stages of the NGT process are described in detail below.

### 2.2 Patient and public involvement and engagement (PPIE)

A group of nine young people who had had stoma surgery, aged between 18 and 37, were recruited via social media advertising to act as advisors to the wider Stoma Support Study of which this consensus study was part. Six of the group are female and three male, located across England and Wales. Three members of the group—M. M. D., S. B. and B. M.—are coauthors on this paper. With regard to the consensus groups study, the advisory group gave input on the study recruitment methods and interpretation of findings. The group also advised on the age range for participants. While initially the focus was on individuals aged 16–29 in line with much of the literature on young adults' experiences of long‐term conditions, the group felt that the upper‐age range should be extended to 35, to reflect the similar challenges people in their early‐to‐mid 30s who have a stoma may be experiencing. As a result of this advice, the participant age range was changed to 16–35.

### 2.3 Participant recruitment

Young people aged between 16 and 35 were recruited through a flyer posted on *Twitter (X)* and shared on stoma and IBD groups/forums on *Facebook*. An advertisement for the study was also shared on the websites and social media channels of national associations for IBD/stoma: Crohn's and Colitis UK, Colostomy UK and the Ileostomy and Internal Pouch Association. We intended to include a varied group of young people; for instance, in relation to age, gender, ethnicity, geographical location and length of time since stoma surgery. While we did not purposively sample for specific characteristics, because of difficulties faced in recruiting individuals from minoritized ethnic groups we did some targeted advertising for people from different ethnic and cultural groups on social media and through the national associations.

Individuals who were interested in participating either contacted the research team directly, via email or *Twitter (X)*, or if they saw the study flyer on the Crohn's and Colitis UK website, they registered their interest on the site and their name and email address were then passed onto the research team. Individuals were then sent a participant information sheet and consent form via email. Having confirmed participation, participants were later emailed to arrange a convenient date and time for the consensus group meeting. Written consent was given via email before meetings, and reaffirmed verbally at the beginning of the consensus group meeting. Before meetings participants were also asked via email to provide information on their gender, location, ethnicity, IBD type and time since surgery. Providing this information was not a prerequisite for participation, and some participants initially did not provide this information and were therefore followed up via email following the consensus group meetings. All participants did ultimately provide demographic information; however, four did not provide information on ethnicity despite being followed up.

### 2.4 Ethics

Ethical approval for the study was granted by the Faculty of Medicine and Health Sciences Research Ethics Committee at Keele University (0038). Participants were reimbursed £50 for taking part, in line with UK NIHR INVOLVE guidance.^22^

#### 2.4.1 NGT meetings

Two online NGT meetings were held in July 2022 with a different group of young people attending each meeting. The groups were run by three members of the research team—B. S.: social science background; K. P.: medical sociology; T. F.: human geography. Before the meetings, participants were emailed evidence maps summarising findings from our qualitative research with young people and HCPs, mentioned earlier^3^, ^16^ (Files SA and SB). The purpose of this was for ‘pre‐elicitation’,^23^ that is, to provide background information and allow participants to start thinking of topics before the meeting.

In meeting 1, following an overview of the study and the NGT process, participants were asked to spend 5 min silently generating topics in response to the question: ‘What areas or topics are most important to include in a support resource to help young people with IBD deal with distress following stoma surgery?’. Participants were then asked to choose 1–2 topics to share with the group in ‘round robin’ fashion, followed by a group discussion of the list of topics generated.

The researchers transferred this list to an online voting platform (www.mentimeter.com). Participants used their mobile phones to access this platform and anonymously rate each topic on a 7‐point Likert scale, in terms of the perceived importance of each for managing stoma‐related distress (7 = *extremely important*; 1 = *not at all important*). Ratings were displayed as the mean across the group, and those topics reaching ≥80%, that is, a mean of ≥5.6/7 on the Likert scale, were considered as reaching consensus. Those with lower than 40%, that is ≤2.8/7, were discarded.

Topics with 41%–79% agreement (2.9–5.5/7) were discussed further, followed by a second round of rating to allow for changes of opinion, with those not reaching 80% consensus discarded after the second vote. Discussion data were used to supplement and contextualise the ratings data but were not formally analysed. The 80% consensus threshold used was based on previous NGT literature which argues that a higher threshold, signalling a stronger level of agreement, indicates more robust evidence for intervention or policy development.^24^

**Figure 1:**
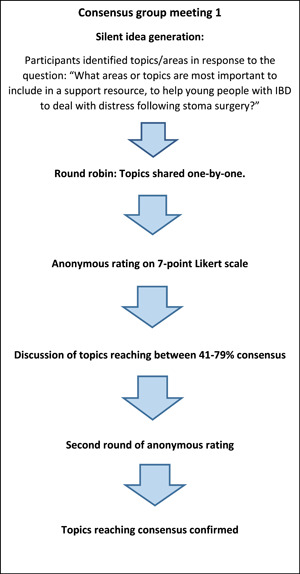
Outline of stages of nominal group technique group 1.

**Figure 2:**
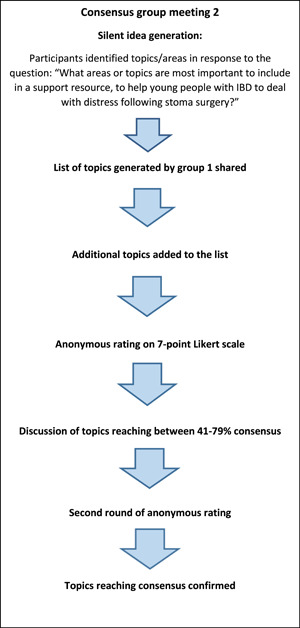
Outline of stages of nominal group technique group 2.

Meeting 2 followed a similar structure, except following the silent topic generation phase, participants were shown the full list of topics generated in meeting 1, and asked to add anything to the list that they felt was missing. Therefore, at the anonymous rating stage the group rated the topics from meeting 1 as well as the additional topics they had proposed. The stages of the two NGT meetings are displayed in Figures 1 and 2.

Following meeting 2, the participants from meeting 1 were recontacted and asked to give ratings for the additional topics generated in meeting 2, on the same 7‐point scale, via email response. This ensured that participants from both groups had rated the same list of topics. The ratings for all topics were then averaged across all participants, and the final percentage of consensus calculated for each topic. Those topics receiving a final mean rating of ≥80% across both groups were confirmed as the priorities for the content of a resource to support young people to self‐manage distress related to stoma surgery.

Following the two consensus group meetings, we held a 2‐h virtual meeting with five members of our PPIE group, in which two members of the research team, B. S. and K. P., discussed the findings with the group, and sought their views on the extent to which the topics that had reached consensus reflected their own views.

In what follows, we present the characteristics of the participant sample followed by results of the consensus group meetings. Quotations from the discussions are presented to contextualise the ratings data:

## 3 RESULTS

### 3.1 Participant characteristics

Nineteen young people with a stoma took part in one of two online NGT meetings (group 1 = 10 participants; group 2 = 9). Fifteen participants were women and four men, aged between 19 and 33, located throughout England, Scotland and Northern Ireland. Nine participants had a stoma as a result of UC, six due to Crohn's disease and one due to indeterminate IBD. The length of time since having stoma surgery ranged from 5 months to 9 years (mean: 3 years, 7 months). Fourteen participants described their ethnicity as White British and one as White Irish. Four participants did not provided information about their ethnicity. Table 1 displays characteristics of the participants separated into groups 1 and 2.

**Table 1:** Characteristics of the participants separated into consensus groups 1 and 2.

Consensus group 1		Consensus group 2	
Age	19–33 years (mean: 25 years)	Age	21−33 years (mean: 27 years)
Gender	8 Women 2 Men	Gender	7 Women 2 Men
Ethnicity	7 White British 1 White Irish 2 Unreported	Ethnicity	7 White British 2 Unreported
Length of time since stoma surgery	Between 1 year and 12 years (mean: 3 years, 10 months)	Length of time since stoma surgery	Between 5 months and 9 years (mean: 3 years)
Location	1 South West England 4 North East England 2 North West England 2 Scotland 1 East of England	Location	1 South East England 1 Northern Ireland 1 Scotland 2 North East England 1 North West England 3 Midlands of England

#### 3.1.1 Priorities identified through the NGT process

##### NGT group 1 results

In meeting 1, 16 topics were proposed to help young people to manage distress following stoma surgery. Following the initial round of anonymous rating, five topics reached the ≥80% consensus threshold. These were as follows

1. Receiving advice from young people with lived experience of stoma surgery.
2. Advice on/addressing concerns about romantic relationships, sex and intimacy.
3. Information about fertility and pregnancy‐related to stoma surgery.
4. Trouble‐shooting guide to the day‐to‐day practicalities of living with a stoma.
5. Stoma ‘hacks’, for example, useful everyday tips regarding clothing to conceal the bag, making bag changes easier, and so forth.

Eleven topics fell between 41%–79%:

1. Show that many aspects of life can return to ‘normal’ following stoma surgery.
2. Representing stoma surgery as a transition rather than a ‘loss’.
3. Advice on wound care.
4. Normalising, but not ‘glamorising’ stomas (i.e., showing relatable images/stories).
5. Addressing concerns about the stoma being outwardly apparent to others, for example, through sound and odour.
6. Distinguishing the management of ongoing IBD symptoms from adapting to the stoma.
7. Advice on disclosing the stoma to others.
8. Information and myth‐busting for family and friends, for example, in relation to misinformation about the impact of food and drink on the stoma.
9. Giving young people goals to work towards after stoma surgery.
10. Advice on practical issues indirectly related to the stoma, for example, in relation to hydration and gum care (removal of the colon puts individuals at increased risk of dehydration. Gum problems associated with Crohn's disease can persist after stoma surgery).
11. Managing stigma and others' reactions to the stoma.

These topics were taken forward for further discussion. There were no topics rated at ≤40%, therefore none were discarded at this stage.

In the discussion of topics rated between 41%–79%, one participant reported their surprise that ‘normalising, but not “glamorising” stomas’ was not rated more highly across the group (mean rating: 3.6/7). They highlighted what they felt was a pervasion of ‘Instagram, essentially models’ *(male participant)* with stomas on social media, which portray unrealistic images that young people may try to emulate. However, the group noted that this topic may overlap with the broader topic of ‘receiving advice from young people with lived experience of stoma surgery’, which may explain its lower rating. The topic ‘show that many aspects of life can return to “normal” following stoma surgery’ (mean: 5.1/7) was also discussed. One participant reported that in their experience, the belief that they would be able to return to ‘normal’ activities following surgery was ‘what got me through in the end’ *(male participant)*. However, others highlighted that returning to normality was not always achievable as some people continue to experience health problems: ‘I was under the false presumption that I would return to “normal”, but unfortunately that's just not how it is for everybody’ *(female participant)*. The group also problematised the notion of ‘normality’, suggesting it may imply ‘blame’ if individuals cannot achieve this. In discussing the topic ‘giving young people goals to work towards after stoma surgery’ (mean: 3.6/7), some participants highlighted that while this topic had merit, it may be difficult to put into practice given people's variable experiences, and that some individuals may never be able to work towards certain goals. One participant suggested more emphasis be placed on acceptance rather than striving to reach new goals: ‘it's focusing more on accepting how things are’ *(female participant)*.

Following the second round of rating, one topic moved above the ≥80% consensus threshold: ‘addressing concerns about the stoma being outwardly apparent to others’; with the remaining 10 topics still falling between 41%–79%, and were therefore discarded. The full ratings data are displayed in Table 2.

##### NGT group 2 results

In meeting 2, 13 new topics were generated, which were added to the list of those generated in meeting 1, making a total of 29 topics. Following the initial round of anonymous rating, five topics reached ≥80 consensus. These were as follows:

1. Receiving advice from young people with lived experience of stoma surgery.
2. Processing trauma related to the illness and surgery journey.
3. Advice about diet and how this may impact on stoma output.
4. Information/advice on having discussions with employers about work adaptations after surgery.
5. Advice on managing skin irritation at the stoma site.

The remaining 24 topics had a mean rating of between 41%–79%, and again no topics were rated ≤40%.

During the discussion of topics rated between 41%–79%, several young people put a case forward that ‘advice on/addressing concerns about romantic relationships, sex and intimacy’ (mean: 4.9/7), should be more highly rated. One participant reported the difficulty in having conversations about sex and intimacy with HCPs, reflecting on ‘really awkward conversations with the stoma nurse and consultant’ *(female participant)*. Others talked about areas in which they felt there was not currently any information available: ‘Just small things, like even when you're having intercourse, the bag, where does it go? What do you do?’ *(female participant)*. The lack of advice for those who are dating or engaging in casual sexual relationships was emphasised: ‘Especially people who are having casual relationships, it can be tricky to navigate to say “I've got a stoma”. So I would have that higher [rated]’ *(female participant)*. The lack of advice available to people in same sex relationships with a stoma was also raised, again particularly in relation to sexual intercourse. Another topic that several participants felt should have been more highly rated was ‘managing stigma and others’ reactions to the stoma' (mean: 5.3/7). One participant highlighted difficult experiences: ‘I've had quite a few people tell me to my face that I am disgusting because I have a stoma’ *(female participant)*, and emphasised the need for advice on managing these types of situations. ‘Information and myth‐busting for family and friends (e.g., in relation to misinformation about the impact of food and drink on the stoma)’ (mean: 5.1/7) was also discussed, with some participants indicating the value that could be had from ‘educating family and friends and making them informed’ *(female participant)*; particularly as it was felt that young people often feel uncomfortable talking to their family about the practical aspects of stoma management.

Following the second round of rating, the following nine topics moved above the ≥80% consensus threshold:

1. Show that many aspects of life can return to ‘normal’ following stoma surgery.
2. Information about fertility and pregnancy related to stoma surgery.
3. Advice on practical issues indirectly related to the stoma, for example, hydration and gum care.
4. Reflecting on/recognising own emotional response to surgery.
5. Tips for managing the stoma during the night.
6. Emphasising positive physical changes postsurgery, for example, greater energy, healing more quickly (faster healing may be a result of individuals no longer needing to take immunosuppressant medication following surgical removal of the colon).
7. Information and ‘myth busting’ for family and friends.
8. Managing stigma and others' reactions to the stoma.
9. Advice on/addressing concerns about romantic relationships, sex, and intimacy.

The remaining 15 topics fell between 41%–79%, and were therefore discarded. The full ratings data are displayed in Table 2.

### 3.2 Calculating consensus across all participants

Following meeting 2, the additional 13 topics generated were sent to participants from group 1 to provide ratings remotely via email. All 10 participants from group 1 responded and provided ratings. Three of these topics reached consensus across this group: reflecting on and recognising own emotional response to surgery; tips for managing the stoma during the night; and processing trauma related to the illness and surgery journey (see Table 2).

The mean scores and percentage consensus for each topic across all 19 participants in the two groups, were then calculated. This resulted in seven topics reaching final consensus, which signal the priorities for self‐management of stoma‐related distress: ‘receiving advice from young people with lived experience of stoma surgery’; ‘advice on/addressing concerns about romantic relationships, sex and intimacy’; ‘information about fertility and pregnancy related to stoma surgery’; ‘stoma “hacks”, for example, useful everyday tips regarding clothing, making bag changes easier and so forth’; ‘reflecting on and recognising own emotional response to surgery’; ‘tips on managing the stoma during the night’; and ‘processing trauma related to the illness and surgery journey’.

Table 2 displays the mean ratings and percentage consensus for each topic in consensus groups 1 and 2, and across all participants. Those topics reaching final consensus are in bold and shaded.

**Table 2:** Mean ratings and percentage consensus for each topic in consensus groups 1 and 2, and across all participants.

Topic generated	Consensus group 1		Consensus group 2		
	Rating round 1	Rating round 2	Rating round 1	Rating round 2	Mean across the 19 participants
Show that many aspects of life can return to ‘normal’ following stoma surgery.	5.1–72.9%	5.2–74.3%	5.3–75.7%	5.7–81.4%	5.3–75.7%
Representing stoma surgery as a transition rather than a ‘loss’.	3.8–54.3%	4–57.1%	3.2–45.7%	3.2–45.7%	3.6–51.4%
**Receiving advice from young people with lived experience of stoma surgery**.	6.6–94.3%	n/a	5.6–80%	n/a	**6.1–87.1%**
**Advice on/addressing concerns about romantic relationships, sex and intimacy**.	6–85.7%	n/a	4.9–70%	6.7–95.7%	**6.3–90%**
**Information about fertility and pregnancy related to stoma surgery**.	5.7–81.4%	n/a	5.4–77.1%	6.2–88.6%	**5.9–84.3%**
Advice on wound care.	4.4–62.8%	3.5–50%	4.7–67.1%	5–71.4%	4.2–60%
Trouble‐shooting guide to the day‐to‐day practicalities of living with a stoma.	6.1–87.1%	n/a	4.8–68.6%	3.7–52.9%	4.9–70%
Normalising, but not ‘glamorising’ stomas' (i.e., showing relatable images/stories).	3.6–51.4%	3.5–50%	3.2–45.7%	3.2–45.7%	3.3–47.1%
Addressing concerns about the stoma being outwardly apparent to others, for example, through sound and odour.	5.4–77.1%	5.6–80%	5.3–75.7%	5.3–75.7%	5.5–78.6%
Distinguishing the management of ongoing IBD symptoms from adapting to the stoma.	4.2–60%	3.8–54.3%	3.3–47.1%	3.3–47.1%	3.5–50%
Advice on disclosing the stoma to others.	4.8–68.6%	4.9–70%	5.1–72.9%	4.9–70%	4.8–68.6%
Information and myth‐busting for family and friends (e.g., in relation to misinformation about the impact of food and drink on the stoma).	5.1–72.9%	4.9–70%	5.1–72.9%	6.2–88.6%	5.5–78.6%
**Stoma ‘hacks’, for example, useful everyday tips regarding clothing to conceal the bag, making bag changes easier, and so forth**.	6.1–87.1%	n/a	5.4–77.1%	5.3–75.7%	**5.7–81.4%**
Giving young people goals to work towards after stoma surgery.	3.6–51.4%	3.3–47.1%	3.6–51.4%	3.6–51.4%	3.4–48.6%
Advice on practical issues indirectly related to the stoma, for example, in relation to hydration and gum care.	5.5–78.6%	4.5–64.3%	5.2–74.3%	5.8–82.9%	5.1–72.9%
Managing stigma and others' reactions to the stoma.	4.9–70%	4.5–64.3%	5.3–75.7%	5.6–80%	5–71.4%
**Reflecting on and recognising own emotional response to surgery**.	5.7–81.4%	n/a	5.4–77.1%	5.7–81.4%	**5.8–82.9%**
Advice about diet and how this may impact on stoma output.	4.6–65.7%	n/a	5.6–80%	n/a	4.9–70%
**Tips for managing the stoma during the night**.	5.6–80%	n/a	5.4–77.1%	5.6–80%	**5.6–80%**
Addressing confidence to leave the house.	5.3–75.7%	n/a	4.2–60%	3.1–44.3%	4.3–61.4%
Making individuals aware of bodily changes indirectly related to stoma, for example hair loss, mucus excrement.	5.1–72.9%	n/a	5.4–77.1%	5.3–75.7%	5.2–74.3%
**Processing trauma related to the illness and surgery journey**.	6–85.7%	n/a	5.9–84.3%	n/a	**5.9–84.3%**
Awareness/signposting to mental health support.	5.2–74.3%	n/a	5.4–77.1%	5.1–72.9%	5.2–74.3%
Adapting to, and accepting, the postsurgery body.	4.8–68.8%	n/a	5.1–72.9%	5.1–72.9%	4.9–70%
Advice on returning to playing sport.	3.6–51.4%	n/a	4.3–61.4%	4.9–70%	4.2–60%
Information for emergency surgery patients, for example, percentage risk of complications.	3.5–50%	n/a	4.6–65.7%	4.6–65.7%	4–57.1%
Information/advice on having discussions with employers about work adaptations after surgery.	4.7–67.1%	n/a	5.6–80%	n/a	5.1–72.9%
Advice on managing skin irritation at the stoma site.	4.7–67.1%	n/a	5.7–81.4%	n/a	5.2–74.3%
Emphasising positive physical changes postsurgery, for example, greater energy, healing more quickly.	5–71.4%	n/a	5.4–77.1%	5.6–80%	5.3–75.7%

Abbreviation: IBD, inflammatory bowel disease.

## 4 DISCUSSION

This is the first study to gain consensus from young people with IBD and a stoma on their priorities for self‐management of stoma‐related distress. We were able to reach a strong level of consensus for seven topics, the highest rated being ‘advice on/addressing concerns about romantic relationships, sex and intimacy’, which reached 90% consensus. Two topics fell just under the consensus threshold: ‘addressing concerns about the stoma being outwardly apparent to others’ and ‘information and myth‐busting for family and friends’ (both 78.6%). It is worth noting that while most topics did not reach the consensus threshold of 80%, the majority of topics did nevertheless still achieve a good level of agreement of 60% or greater. This may suggest that many of the challenges faced are common between young people with a stoma, as well as reflecting the homogeneity of this group in terms of age and condition. For instance, a generalised age group of people with a stoma may have reached lower levels of consensus, due to having different needs and priorities related to their respective life stages.

These results were discussed with our PPIE group, and while the group were in agreement with the priorities that had reached consensus, they also picked out two topics that they felt were important, but which had not reached consensus: ‘awareness/signposting to mental health support’ and ‘addressing concerns about the stoma being outwardly apparent to others’. Some of the PPIE group reflected that, for them, concerns about the outward appearance of the stoma was a major source of anxiety after stoma surgery.

These findings show some similarity with previous research, including our earlier research on young people's experiences of stoma surgery, which identified similar issues such as fear of stigmatising reactions and concerns about romantic relationships.^3^ Earlier studies by Savard and Woodgate^25^ and Allison et al.,^14^ both similarly highlighted young people's dissatisfaction about physical appearance resulting in concerns about appearing unattractive to romantic partners. A more recent qualitative study with 43 people with IBD, mainly from the United Kingdom, 11 of whom also had a stoma, highlighted that people with IBD saw sex as important within their lives. However, they reported facing challenges due to their condition, and subsequently experienced interruptions to intimacy and sexual relationships and negative impacts on sexual wellbeing.^26^ It was also reported that individuals' concerns about sex were not being discussed in healthcare settings, or when concerns were raised by people with IBD to their HCPs, these were not adequately addressed.^27^ An Irish study in which five young adult men, aged 20–30, were interviewed about their experiences of IBD and a stoma similarly reported a negative impact of their stoma on a few of the men's sexual relationships. This was primarily due to their concerns that sexual partners may react negatively to the stoma.^28^ In the present study, the topic of romantic relationships did not directly relate to dissatisfaction about physical appearance or concerns about sexual activity being negatively impacted due to partners' reactions. Rather, participants highlighted the need for young people to be given practical information and advice about being intimate with partners, for example, the practicalities of sexual intercourse (including for those in same sex relationships), as well as advice about how to broach the issue with potential romantic partners or in casual sexual relationships or encounters. The issue of how to manage the stoma and share information in casual sexual encounters has not, to our knowledge, been reported in previous literature.

Comparisons can also be made with research on more generalised age groups. A recent UK‐based Delphi study by Aibibula et al.^29^ aimed to reach consensus on the difficulties faced by people with a stoma, aged 26–75, particularly focusing on stoma bag leaks. Through this, a ‘Call to Action’ was developed to improve stoma care in clinical settings, which included: delivery of individualised and holistic stoma care, access to mental health support, and an annual review that includes mental health, skin health and product choice. While the emphasis on addressing the emotional and psychological aspects of stoma management is common with our findings, concern about the stoma bag leaking was not an issue raised by our participants. Also, several of the priority areas that reached consensus in our study were not identified by Aibibula et al.^29^ This included ‘information about fertility and pregnancy related to stoma surgery’. Some female participants reported concerns they had felt following surgery that they may have decreased fertility, which may reflect a priority that is specific to younger people with a stoma, and perhaps younger women in particular. However, a recent Cochrane review concluded that the effects of surgery for IBD on female infertility and pregnancy‐related outcomes is uncertain, due to poor quality of existing literature,^30^ and therefore this is an area in need of further research.

### 4.1 Strengths and limitations

The NGT method used is a strength of the study. In particular, the anonymous rating allowed for each participant's views to be given equal weighting in the results. Another strength is that the topics that were rated and discussed were generated by participants themselves, enabling findings to reflect issues that were important to the young people.

A limitation is the lack of variation in the participant sample. Three quarters of the participants were women, and there was a lack of ethnic and cultural diversity. This meant that the views and priorities of young people from Black, Asian and Minority Ethnic communities were not represented in the findings, reflecting broader challenges in recruiting young people from these groups to healthcare research.^31^, ^32^ An important area for future research is therefore to understand the needs and priorities of young people with a IBD and a stoma from diverse cultural and ethnic backgrounds. We are actively addressing this issue ahead of the next phase of the study by conducting PPIE work with individuals from diverse communities, through which we aim to build relationships and gain advice on how to best engage with young people with an IBD stoma from minoritized ethnic backgrounds.

## 5 CONCLUSIONS AND IMPLICATIONS

The findings extend previous research exploring young people's concerns following stoma surgery, by systematically identifying and gaining consensus on priority topics for self‐management of distress related to surgery and living with a stoma. Seven topics reached a strong level of consensus, some of which indirectly target distress through addressing stoma‐related challenges, for example, advice on managing the stoma during the night; while others focus directly on self‐managing distress, for example, processing trauma related to the illness and surgery journey. Some of the areas agreed are those which may be particularly hard for young people to raise with their HCPs for example, sex and intimacy. This may highlight the importance of resources including ‘peer’ support, which represents the voices of other young people who can talk from experience.

These findings will directly inform the development of a digital self‐management resource, that will be co‐produced with young people with a IBD and a stoma, and HCPs, through a series of development workshops. Involving young people in the development of support resources, using methods which enable them to have an equal say in what matters to them, is vital to ensure that the needs of the young people are being met.^33^ This resource, along with the other part of the intervention—a brief online training package for HCPs on identifying and managing stoma‐related distress^21^—has the potential to reduce distress in young people with IBD and a stoma, and thereby improve their quality of life and clinical outcomes. The findings also have direct implications for healthcare provision. Making primary and secondary care professionals aware of these priorities may encourage them dedicate more time to addressing these in consultations, which can enhance the clinical management of distress in this population.

## AUTHOR CONTRIBUTIONS

**Benjamin Saunders**: Conceptualization; data curation; formal analysis; funding acquisition; investigation; methodology; project administration; writing—original draft; writing—review & editing. **Kay Polidano**: Conceptualization; data curation; formal analysis; funding acquisition; investigation; methodology; writing—review & editing. **Lucy Bray**: Conceptualization; data curation; formal analysis; funding acquisition; investigation; methodology; writing—review & editing. **Tamsin Fisher**: Conceptualization; data curation; formal analysis; funding acquisition; investigation; methodology; writing—review & editing. **Nadia Corp**: Conceptualization; formal analysis; funding acquisition; investigation; methodology; writing—review & editing. **Megan McDermott‐Hughes**: Formal analysis; investigation; methodology; writing—review & editing. **Adam D Farmer**: Conceptualization; formal analysis; funding acquisition; investigation; methodology; writing—review & editing. **Beth Morris**: Formal analysis; investigation; methodology; writing—review & editing. **Sahara Fleetwood‐Beresford**: Formal analysis; investigation; methodology; writing—review & editing. **Carolyn A Chew‐Graham**: Conceptualization; formal analysis; funding acquisition; investigation; methodology; writing—review & editing.

## CONFLICT OF INTEREST STATEMENT

Professor Carolyn Chew‐Graham is Editor‐in‐Chief of Health Expectations. The other authors declare that they have no conflicts of interest.

## ETHICS STATEMENT

Ethical approval for the study was granted by the Faculty of Medicine and Health Sciences Research Ethics Committee at Keele University (0038). All participants provided written informed consent to take part in the study, and for their anonymised quotes to be presented in the manuscript.

## Supporting information

Supporting information.

Supporting information.

## Data Availability

In line with the Standard Operating Procedures in place at Keele School of Medicine, where this study was conducted, data are archived at a dedicated location within the Keele University's network. A request to access archived data can be made by completion of a Data Transfer Request form, which can be accessed by contacting: Primary Care Centre Versus Arthritis, School of Medicine, Keele University, Staffordshire, ST5 5BG, UK; Tel: +44 (0) 1782 733905.
