# Supplementary material for: Young people's priorities for the self‐management of distress after stoma surgery due to inflammatory bowel disease: A consensus study using online nominal group technique
Source: Health Expect. 2024 Mar 10;27(2):e14009. doi: 10.1111/hex.14009 (PMC10925815; doi:10.1111/hex.14009)
Supplement: Supplementary file 1 — Supporting information. [file HEX-27-e14009-s001.pdf]

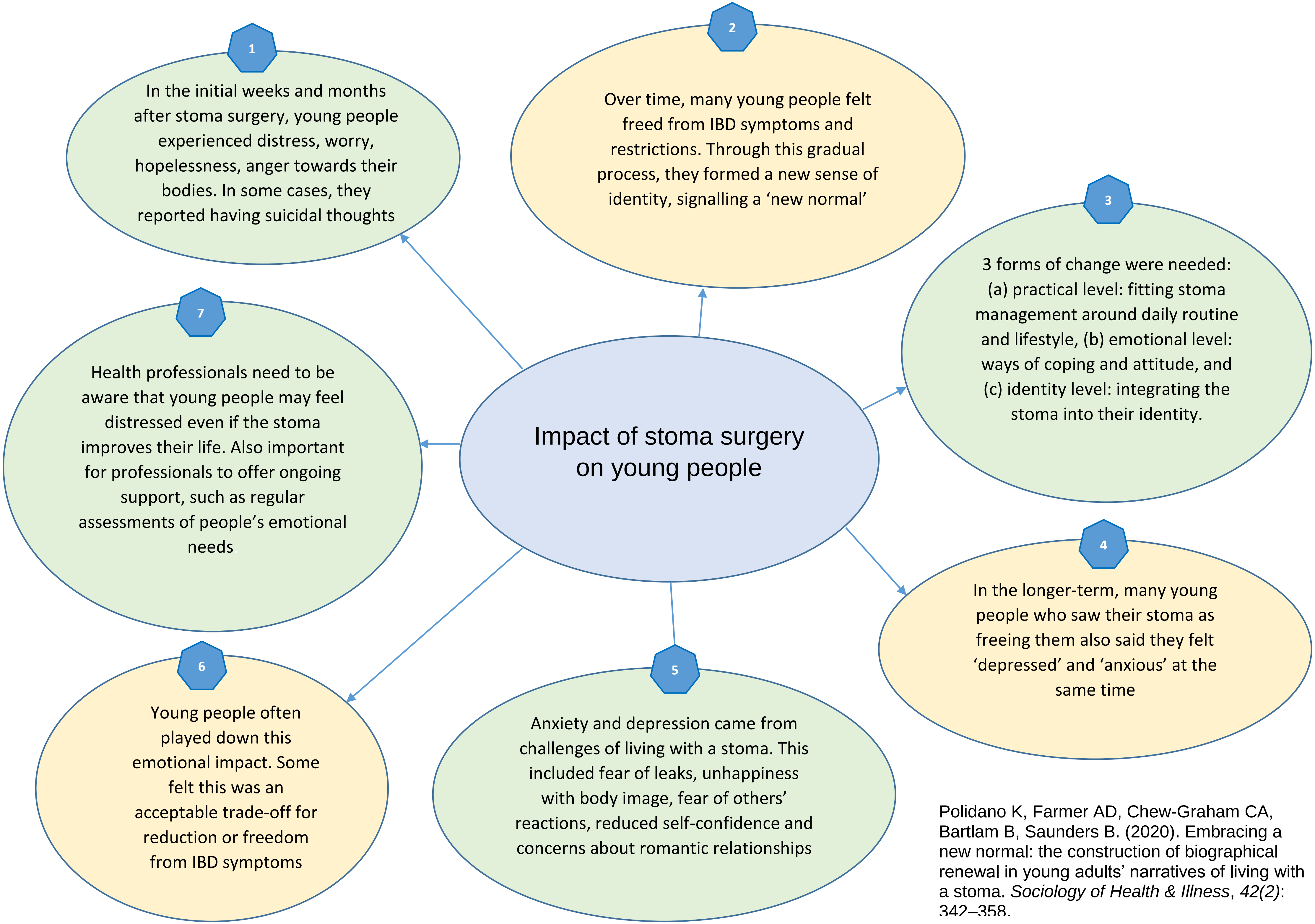

Polidano K, Farmer AD, Chew-Graham CA, Bartlam B, Saunders B. (2020). Embracing a new normal: the construction of biographical renewal in young adults' narratives of living with a stoma. *Sociology of Health & Illness*, *42(2)*: 342–358.
