# Supplementary material for: Young people's priorities for the self‐management of distress after stoma surgery due to inflammatory bowel disease: A consensus study using online nominal group technique
Source: Health Expect. 2024 Mar 10;27(2):e14009. doi: 10.1111/hex.14009 (PMC10925815; doi:10.1111/hex.14009)
Supplement: Supplementary file 2 — Supporting information. [file HEX-27-e14009-s002.pdf]

Help for distress following stoma surgery

Findings from interviews with: People aged 18-29; GPs; Gastroenterologists; IBD and Stoma nurses; Surgeons

Beginning help-seeking

- Some young people felt that their emotional needs didn't deserve professional attention. They felt that negative emotions were a normal reaction to having stoma surgery
- People who looked for support said there was a lack of information about support services. They felt lost trying to find their way around the care system
- Young people were most likely to tell their GP about emotional concerns, followed by their IBD nurse, then stoma nurse. The relationship with the professional was important in their decision about who to talk to
- Stoma nurses saw identifying distress and worries as a key part of their role. Consultants also felt stoma nurses were the best group to do this
- Young people said that stoma nurse appointments usually focused on practical aspects of stoma care. They felt that there wasn't time to bring up emotional concerns

Assessing distress and mental health needs

- GPs were the most confident group in assessing mental health needs, but felt they lacked specialist knowledge about stomas
- Professionals found it difficult to decide if young people had symptoms of depression, or if they were having a "normal" response to surgery
- When professionals described distress as "normal", patients sometimes felt they weren't being taken seriously

Providing support for distress and mental health needs

- Stoma nurses highlighted a lack of formal training in managing psychological issues. They said they would sometimes refer young people to specialist services
- Patients were often told to see their GP about distress. GPs often suggested the young person contact local psychological therapy services. However, young people felt these services lacked knowledge about stomas
- The lack of psychological support as part of the IBD Service made it difficult for health professionals to help their patients to access support
- Young people and health professionals highlighted the need for a psychological service specifically for IBD patients after stoma surgery

Polidano K, Chew-Graham C, Farmer AD, Saunders B. (2020). Access to psychological support for young people following stoma surgery: exploring patients' and clinicians' perspectives. *Qualitative Health Research.* 31(3): 535-49.
